# Liquid Biopsy for Cerebral Aneurysms: Circulating RNA as Diagnostic and Prognostic Tools—A Systematic Review of Current Evidence and Perspectives

**DOI:** 10.3390/cells14191525

**Published:** 2025-09-30

**Authors:** Matteo Palermo, Alessandro Olivi, Carmelo Lucio Sturiale

**Affiliations:** Department of Neurosurgery, Fondazione Policlinico Universitario A.Gemelli IRCCS, Università Cattolica del Sacro Cuore, Largo A. Gemelli 8, 00168 Rome, Italy; matteo.palermo01@icatt.it (M.P.); alessandro.olivi@policlinicogemelli.it (A.O.)

**Keywords:** intracranial aneurysm, liquid biopsy, microRNA, lncRNA, circRNA, biomarker

## Abstract

Intracranial aneurysms (IAs) are potentially devastating cerebrovascular lesions, and predicting rupture risk remains a major clinical challenge. Conventional radiological and clinical scores offer only partial risk stratification, highlighting the need for complementary approaches. Liquid biopsy represents a promising non-invasive strategy to identify circulating biomarkers that reflect aneurysm biology and instability. We conducted a systematic review according to PRISMA 2020 guidelines, screening PubMed, Scopus, and Web of Science up to August 2025. Forty-eight eligible studies, encompassing 3515 IA patients, evaluated circulating RNA species, including microRNAs (miRNAs), long non-coding RNAs (lncRNAs), and circular RNAs (circRNAs) in serum, plasma, blood, or cerebrospinal fluid. Multiple candidates emerged as consistently dysregulated: upregulation of miR-21, miR-126, and miR-200a-3p, and downregulation of miR-143 and let-7b-5p were recurrently observed across independent cohorts. LncRNAs, such as MALAT1 and MIAT, and circRNAs, including circ_0000690 and circ_0021001, demonstrated diagnostic and prognostic potential, with some correlating with rupture status and clinical severity indices. Despite encouraging findings, heterogeneity in study design, sample handling, and analytic methods limits reproducibility. Large-scale, multicenter validation studies are essential to translate these biomarkers into clinical practice.

## 1. Introduction

In oncology, the term liquid biopsy refers to sampling circulating biomarkers from blood to non-invasively gather information about a tumor’s molecular characteristics [1,2,3,4,5,6,7]. Potentially, liquid biopsy may provide information regarding the diagnosis and prognosis of oncologic patients, also offering targets for chemotherapy.

Inspired by this concept, there has been increasing interest in identifying circulating biomarkers linked to intracranial aneurysms (IAs) [8]. In particular, markers reflecting inflammation or extracellular matrix (ECM) remodeling have been associated with endothelial injury, which may contribute to IA formation and rupture. However, their expression is strongly influenced by systemic factors, making them non-specific to IA pathophysiology. For this reason, unlike in neuro-oncology, a comparable approach of “vascular liquid biopsy” has not yet been integrated into clinical practice to assess blood or cerebrospinal fluid (CSF) for IA-related biomarkers [1].

Among the potential candidates, the circulating genetic material has emerged as particularly promising to detect the presence of IAs or to estimate rupture risk. Yet, despite its potential, no validated screening protocol is currently available, and this field remains rather unexplored.

Today, the natural history and rupture risk of IAs are not yet fully understood. The ISUIA trial showed that rupture risk varies depending on size and location, but it is typically low in unruptured IAs [9]. For ruptured aneurysms, the ISAT compared endovascular coiling to surgical clipping and discovered that coiling reduced morbidity and mortality, profoundly influencing the treatment approaches around the world [10].

In addition to these studies, a number of scoring systems that incorporate both angioarchitectural and patient-specific risk factors have been developed to improve rupture risk assessment. Among them, in order to predict rupture risk, the PHASES score evaluates variables like aneurysm size, location, and patient history, including age and hypertension. The ELAPS score takes into account the size, shape, and age of aneurysms in addition to hypertension [11]. Additionally, in order to support treatment decisions for unruptured IAs, a multi-factor, consensus-based tool called UIATS was built, combining imaging and clinical information [12,13,14]. However, clinical decision-making is only in part sustained by these systems, which may help in identifying patients who are more likely to rupture [12,14,15], but the final clinical judgement is still under the responsibility of the institutional neurovascular team and the agreement with patient volunteers after the informed consent.

Current management has been only partially aided by these instruments, as well as cutting-edge imaging techniques like vessel wall MRI [16], and a definitive screening protocol for IA incidence and risk of rupture does not yet exist in the general population.

Therefore, it would be extremely valuable to identify reliable circulating biomarkers acting as a liquid biopsy, providing non-invasive diagnostic and prognostic information about IAs, thereby improving early detection, risk stratification, and patient management. In this scenario, circulating genetic material offers a novel chance for “vascular liquid biopsy”, but its clinical translation is still in its beginning.

This systematic review aims to summarize current evidence and highlight future directions in this emerging field.

## 2. Methods

**Figure 1:**
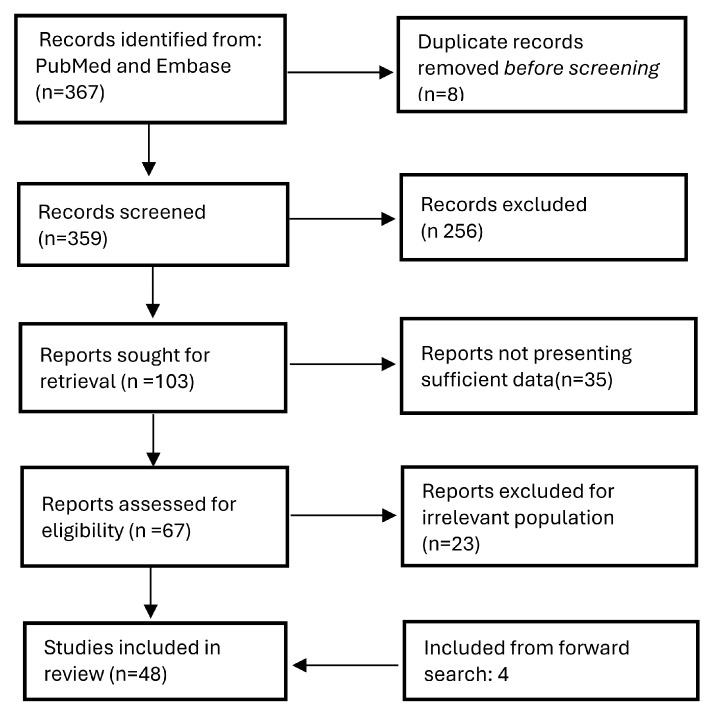
PRISMA flowchart of study selection process.

This review was performed according to the PRISMA (Preferred Reporting Items for Systematic Reviews and Meta-Analyses) 2020 guidelines [17]. To structure the research question, we employed the PICO framework, defining the population as patients with ruptured or unruptured intracranial aneurysms, the intervention as biomarker testing (RNA), the comparison group as controls, and the outcome as changes in biomarker expression, assessed by up- or down-regulations (Figure 1).

### 2.1. Search Strategy

Two authors (C.L.S. and M.P.) conducted a literature search using the PubMed/MEDLINE and Scopus databases to identify studies measuring the levels of circulating biomarkers in patients presenting with an IA. The search strategy employed the following string: “(circRNA OR circular RNA OR miRNA OR microRNA OR lncRNA OR long non-coding RNA OR exosom*) AND (intracranial OR brain) AND (aneurysm*)”. The search was last updated on August 30th, 2025, with no date restrictions. We also performed a forward search from the included studies.

### 2.2. Study Selection

Only peer-reviewed studies published in English and reporting quantitative data on circulating genetic biomarkers, and in particular RNA, in body fluids of patients with IAs were considered eligible. Studies analyzing biomarkers from tissue biopsies were excluded, as we specifically focused on circulating genetic material already tested in human fluids and applicable as a liquid biopsy approach. Additional exclusion criteria were animal or pre-clinical studies, review articles, and papers lacking quantitative results. Studies assessing predefined biomarker panels for IA presence or rupture were excluded, as their predictive performance may reflect the combined contribution of multiple markers, thereby obscuring the individual predictive value of each biomarker. We also excluded studies that did not report specific information on the status of expression of the biomarkers. However, an exception was made when a single marker within a panel demonstrated outstanding prominence with statistically significant relevance. Finally, studies limited exclusively to familial or multiple IA forms were not included.

Two authors (CLS and MP) independently screened the titles and abstracts of all retrieved articles, followed by full-text screening of studies that either met the inclusion criteria or had uncertain eligibility (Figure 1).

We included only RNA biomarkers suitable for vascular liquid biopsy while excluding systemic inflammatory markers, as these cannot be considered specific for the presence or rupture of IAs when assessed individually rather than in combination.

### 2.3. Data Extraction

For each study included in this systematic review, we recorded the first author and year of publication (Table 1). In addition, we extracted data on the number of patients tested and the expression patterns of the investigated biomarkers. For all cases, we also documented the biomarker name, its type and family, and the liquid source from which it was obtained.

### 2.4. Risk of Bias

**Figure 2:**
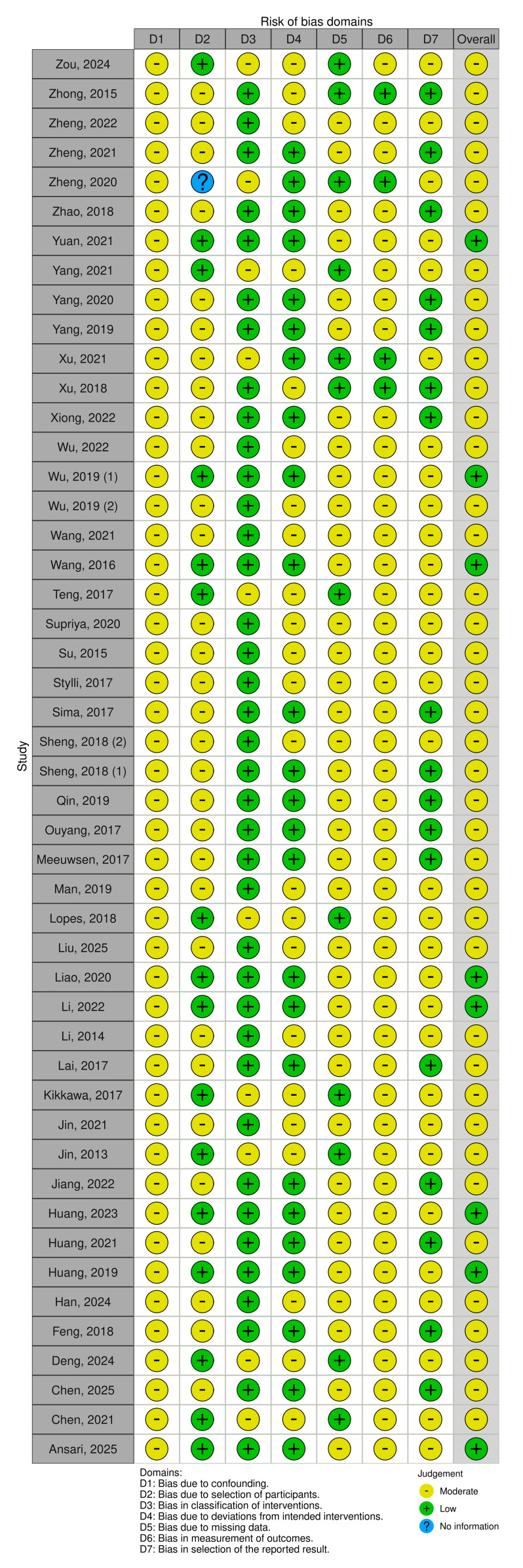
ROBINS-V2 tool for publication bias of the selected articles [8,18,19,20,21,22,23,24,25,26,27,28,29,30,31,33,34,35,36,37,38,39,40,41,42,43,44,45,46,47,49,50,51,52,53,54,55,56,57,59,60,61,62,63,64,65].

The ROBINS-I V2 (Risk of Bias in Non-randomized Studies of Interventions, Version 2) tool was used to assess the methodological quality of the included studies. The results of this evaluation were then visualized using the robvis web application (https://mcguinlu.shinyapps.io/robvis/ accessed on 28 August 2025), which generates illustrative summaries of risk of bias assessments (Figure 2).

## 3. Results

The initial search algorithm yielded 367 records. During the initial screening phase, we excluded 20 non-English articles, 8 duplicates, 112 reviews, 15 articles not related to biomarkers, 63 studies that did not address the target population, and 46 articles as they were not pertinent to the research topic.

Following this preliminary selection, we carried out a full-text screening phase. At this time, we excluded 36 articles due to insufficient or irrelevant data and 23 for not dealing with the selected population (Figure 1, Table 1). Four articles were included from the forward search.

Overall, 48 studies were included in this systematic review. The study selection process adhered to the PRISMA 2020 guidelines (Figure 1). The ROBINS-I V2 tool was used to assess the risk of bias for each included study (Figure 2).

**Figure 3:**
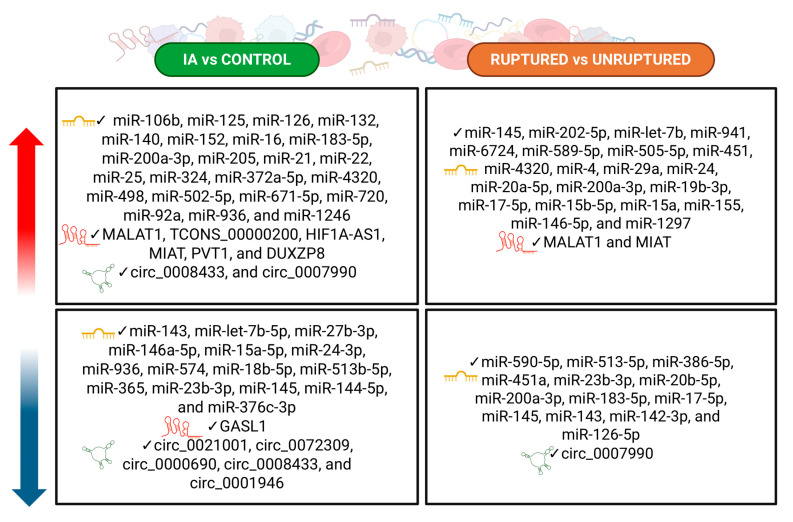
Graphical summary of biomarkers up- and down-regulated for IA vs. Control and Ruptured vs. Unruptured status.

A total of 48 human studies published between 2013 and 2025 were included in this systematic review, investigating 97 unique RNA biomarkers in 3415 IA patients [8,18,19,20,21,22,23,24,25,26,27,28,29,30,31,33,34,35,36,37,38,39,40,41,42,43,44,45,46,47,49,50,51,52,53,54,55,56,57,59,60,61,62,63,64,65]. The majority focused on circulating miRNAs (82/97, 83.7%), with fewer exploring lncRNAs (8/97, 8.1%) and circRNAs (9/97, 9.2%). Overall, 68.3% (67/97) of the studies compared IA patients with healthy controls, while 44.9% (44/97) evaluated differences between ruptured and unruptured aneurysms (Table 1, Figure 3).

Across studies investigating biomarkers for IAs, several candidate miRNAs have been identified. We classified these miRNAs into two groups, upregulated and downregulated, according to both their occurrence and their association with rupture status.

Specifically, miR-106b, miR-125, miR-126, miR-132, miR-140, miR-152, miR-16, miR-183-5p, miR-200a-3p, miR-205, miR-21, miR-22, miR-25, miR-324, miR-372a-5p, miR-4320, miR-498, miR-502-5p, miR-671-5p, miR-720, miR-92a, miR-936, and miR-1246 were consistently reported as upregulated [18,19,20,21,25,29,35,40,41,42,43,44,49,52,57,59,61,62].

In contrast, others, such as miR-143, miR-let-7b-5p, miR-27b-3p, miR-146a-5p, miR-15a-5p, miR-24-3p, miR-936, miR-574, miR-18b-5p, miR-513b-5p, miR-365, miR-23b-3p, miR-145, miR-144-5p, and the cluster miR-376c-3p, were uniformly downregulated [18,22,23,28,33,34,41,42,46,54,55,58].

Notably, the expression profiles of miR-29a-3p [22,33,34,41,64], miR-34a-5p [42,47,58], and miR-145 [28,33,34,43] showed conflicting results depending on the study.

Seven studies assessed lncRNAs showing mainly increased expression of MALAT1, TCONS_00000200, HIF1A-AS1, MIAT, PVT1, and DUXZP8 [19,30,31,49,61,62], with GASL1 as the only lncRNA reproducibly downregulated [39].

Seven studies investigated circRNAs in IA patients, with the majority reporting decreased expression levels (circ_0021001, circ_0072309, circ_0000690, circ_0008433, and circ_0001946) [8,24,40,56]. However, some circRNAs, such as circ_0008433 and circ_0007990, were found to be upregulated [37,40,51].

With respect to rupture status, several miRNAs, including miR-145, miR-202-5p, miR-let-7b, miR-941, miR-6724, miR-589-5p, miR-505-5p, miR-451, miR-4320, miR-4, miR-29a, miR-24, miR-20a-5p, miR-200a-3p, miR-19b-3p, miR-17-5p, miR-15b-5p, miR-15a, miR-155, miR-146-5p, and miR-1297, were reported at higher levels in ruptured compared to unruptured IAs [26,27,30,31,32,38,43,45,55,60,62].

Conversely, other markers such as miR-590-5p, miR-513-5p, miR-386-5p, miR-451a, miR-23b-3p, miR-20b-5p, miR-200a-3p, miR-183-5p, miR-17-5p, miR-145, miR-143, miR-142-3p, and miR-126-5p were found to be downregulated [8,22,23,30,33,41,55]. Notably, miR-29a-3p gave discordant results across studies [22,45,62]. Among lncRNAs, only MALAT1 and MIAT showed consistent upregulation with rupture [29,60], whereas circRNAs generally declined in ruptured IAs, except for circ_0007990, which was increased [51].

Biofluid sources were heterogeneous across studies. Serum represented the most frequently used matrix (55%, n = 33), followed by plasma (20%, n = 12), whole blood (18.3%, n = 11), and CSF (6.7%, n = 4), the latter exclusively analyzed in ruptured aneurysm patients.

## 4. Discussion

**Figure 4:**
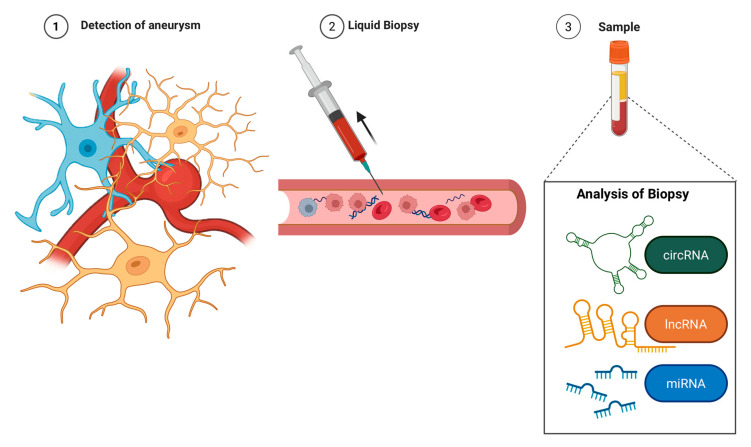
Liquid biopsy for intracranial aneurysm.

Circulating nucleic acids RNA species have gained attention as minimally invasive “liquid biopsy” biomarkers for IAs detection and risk of rupture stratification (Figure 4).

### 4.1. MicroRNAs

Circulating microRNAs have emerged as promising minimally invasive biomarkers for IAs, although findings across studies remain partly inconsistent. Multiple independent studies have detected upregulation of certain miRNAs in IA patient cohorts, suggesting a robust association with the disease. For instance, miR-21, a regulator of inflammatory pathways, has been consistently higher in the blood of IA patients [48,49], echoing earlier observations in smaller cohorts [18]. In the same vein, early microarray-based screens by Li et al. in 2014 and Su et al. in 2015 identified additional miRNAs (miR-16, miR-25, miR-132, and miR-324) as upregulated in the IA patients’ plasma, providing a foundation for subsequent targeted investigations [19,20]. Similarly, the vascular remodeling-associated miR-200a-3p was elevated in both a small initial series and a recent multi-fluid analysis [23,62]. Another pro-inflammatory miRNA, miR-155, was found to be increased in a large patient cohort by Yang et al., published in 2019, reinforcing its putative role in aneurysm pathogenesis [38]. In addition to these, other upregulated candidates have been reported, such as miR-92a [48], miR-125a [53], miR-126 [64], and miR-205 [21], though each of these has so far been documented by a single group.

Conversely, a number of circulating miRNAs appear consistently downregulated in IAs. Notably, members of the miR-143/145 family, which are involved in maintaining vascular smooth muscle cell phenotype, have been observed at lower levels in IA patients’ blood across several studies [28,33,34]. Likewise, miR-23b-3p has been found to be reduced in IA patients [55,63], pointing to a recurring pattern of decreased *“protective”* miRNAs in the disease.

However, another study by Liao et al. in 2020 focused on exosomes, revealing an opposite trend for miR-145-5p that was found upregulated in plasma exosomes from IA patients, with levels even higher in cases of ruptured IAs [43]. This general downregulation is consistent with the protective roles of these miRNAs in the vasculature. The disparity between the exosomal results of Liao et al. and the decreases in total plasma observed by other authors [34] highlights how variations in specimen type and methodology (isolated exosomes vs. whole serum/plasma) can affect the detection of biomarkers.

A similar pattern of conflicting results has been observed with miR-29a-3p, another key regulator of extracellular matrix turnover. Zhao et al. in 2018 and Liao et al. in 2020 documented higher miR-29a-3p in circulating exosomes from IA patients [35,43]. Notably, already in 2016, Wang et al. had reported this miRNA to be significantly decreased in plasma in a larger cohort, including lower levels in patients with ruptured aneurysms [22]. Intriguingly, when examined in the CSF, miR-29a-3p was again found elevated in ruptured IA cases [45], hinting that compartmental differences (blood vs. CSF) may also contribute to the divergent observations.

These examples illustrate how cohort variability, biofluid type, and technical approaches may lead to apparently discordant results in the miRNA biomarker literature. Additional instances of inconsistent findings include miR-34a-5p and miR-146a-5p. Two groups observed miR-34a downregulated in IA patients’ serum [47,58], suggesting that its loss might be associated with IAs’ presence, yet another study identified miR-34a-5p as elevated [42]. Similarly, one IA cohort by Lopes et al. [30] showed an increase in the inflammation-linked microRNA miR-146a-5p [30], while another population showed a decrease [42]. These discrepancies most likely result from variations in detection platforms, sample handling, and patient demographics or inflammatory status. It should be noted that some of these disparities resulted from high-throughput screening initiatives. For instance, Supriya et al. (2020) identified a panel of dysregulated miRNAs using a broad microarray-based approach, some of which were not supported by later targeted analyses [42]. This emphasizes how crucial validation is and how discovery techniques may affect preliminary findings.

### 4.2. LncRNAs

Beyond miRNAs, circulating lncRNAs have also shown diagnostic and prognostic relevance in IAs. Using microarray screening and qRT-PCR validation, Wu et al. demonstrated that four circulating lncRNAs were significantly dysregulated in IA patients compared to healthy individuals [37]. Among them, plasma lncRNA TCONS_00000200 was markedly elevated (2.3-fold) in IA patients, distinguishing aneurysm carriers with 90% sensitivity and 96.7% specificity (AUC = 0.963), thereby underscoring its potential as a robust non-invasive biomarker [37]. Similarly, other circulating lncRNAs such as MALAT1 and MIAT have been identified as clinically relevant: MALAT1 expression was significantly upregulated in IA patients and independently associated with hypertension, rupture status, Hunt–Hess grade, and poor overall survival, suggesting its value as a prognostic indicator [29,60]. MIAT was also found to be elevated in serum, with higher levels in ruptured compared to unruptured aneurysms [66]. Crucially, MIAT expression was found to be an independent risk factor for rupture and a poor long-term prognosis, and it was able to distinguish between healthy people, UIA, and RIA cases (AUCs ranging from 0.69 to 0.79).

Mechanistic research indicates that lncRNAs play functional roles in the pathophysiology of IAs in addition to their diagnostic potential. PVT1 promotes IAs development by activating pyroptosis in cerebral smooth muscle cells through the USP10/KLF4/NLRP3 axis, increasing IL-1β, IL-18, and caspase activity, thereby linking inflammation and vascular remodeling to IAs progression [61]. Exosomal lncRNAs also appear to contribute: serum-derived DUXAP8 was shown to regulate CHPF2 levels, enhancing endothelial inflammation and IAs progression [57]. Its diagnostic utility was supported by ROC analyses (AUC 0.77–0.94), indicating that extracellular vesicle-borne lncRNAs may serve as highly accessible biomarkers.

Furthermore, downregulation of the lncRNA GASL1 was observed in IA patients, inversely correlating with serum TGF-β1 levels [39]. Functional assays showed that GASL1 overexpression enhanced vascular smooth muscle cell proliferation while suppressing TGF-β1, highlighting a potential protective role against aneurysm wall degeneration [39].

Taken together, these studies demonstrate that circulating lncRNAs, whether free or vesicle-derived, not only reflect the presence and rupture risk of intracranial aneurysms but also actively participate in disease mechanisms.

### 4.3. CircRNAs

Emerging evidence has increasingly implicated circRNAs, covalently closed RNAs with exceptional stability, as novel biomarkers in IAs. Several studies have consistently reported dysregulated circRNAs in both aneurysm tissues and circulation. For instance, hsa_circ_0000690 has been identified as a promising circulating biomarker: its expression is significantly reduced in IA patients compared with healthy controls, achieving diagnostic accuracy with an AUC of 0.752, 78% specificity and 62% sensitivity [56]. Moreover, low levels of hsa_circ_0000690 correlated with clinical severity indices, such as Glasgow Coma Scale, Fisher grade, Hunt–Hess score, and hemorrhage volume, and predicted worse 3-month functional outcome after surgery [56].

Other circRNAs have also been implicated. Hsa_circ_0021001 was found to be downregulated in IA patients’ blood, with strong diagnostic performance (AUC = 0.87) and associations with rupture status, Hunt–Hess grade, and timing of surgery [24]. Higher expression levels correlated with improved disease-free and overall survival, suggesting both a possible diagnostic and prognostic relevance. Similarly, hsa_circ_0008433 and hsa_circ_0001946 were independently associated with IAs rupture risk, and reduced expression increased susceptibility, especially in synergy with aging [8,67]. Additionally, high-throughput sequencing studies have identified hundreds of circRNAs differentially expressed in IAs’ walls compared to controls, with circ_0072309 showing consistent alterations in both central tissues and peripheral blood, reinforcing their biomarker potential [40].

Mechanistically, circRNAs may regulate vascular pathology through competitive endogenous RNA (ceRNA) networks, acting as sponges for microRNAs and influencing downstream gene expression in processes such as smooth muscle cell phenotypic switching, extracellular matrix remodeling, and inflammation, all central to IA pathogenesis [24,51,67]. This functional relevance strengthens their candidacy as diagnostic tools. Notably, hsa_circ_0005505 has been shown to promote vascular smooth muscle cell proliferation and migration while inhibiting apoptosis, potentially contributing to aneurysm growth and rupture [50].

Furthermore, circRNAs may aid in stratifying IA subgroups: for example, hsa_circ_0007990 was significantly upregulated in patients with unruptured IAs exhibiting enhancement of the vessel wall on MRI, suggesting a role as an inflammatory biomarker linked to wall instability [51].

### 4.4. Potential Clinical Applications

Although current evidence on RNA biomarkers in IAs is still preliminary, several potential clinical applications are emerging. The concordant expression of specific circulating miRNAs like miR-21 [18,23,48,49], miR-126 [30,64], miR-200a-3p [23,62], miR-143 [28,33,34], miR-let-7b-5p [23,30,45], miR-24-3p [42,45], and the lncRNAs MALAT1 and MIAT [29,60,66] across different studies suggests that these molecules could be considered candidate diagnostic markers. However, given the modest statistical associations and the small sample sizes, it is not yet feasible to speculate about reliable risk-stratification tools; thus, these findings should be regarded as preliminary markers of interest requiring validation in larger prospective studies.

Nevertheless, the fact that several biomarkers were reproduced across independent studies strengthens confidence in their potential value. Thus, at present, circulating RNAs should be interpreted as candidate risk factors, with future large-scale validation needed before they can be deployed as definitive markers or integrated into standardized screening and management protocols [21,67].

At present, clinical decision-making for unruptured intracranial aneurysms primarily relies on risk scores such as PHASES and UIATS, supported by advanced imaging tools, including vessel wall MRI. If validated in large prospective cohorts, in the near future, it is conceivable that standardized genetic panels incorporating circulating RNAs could be developed, enabling rapid and user-friendly laboratory screening for IA risk.

### 4.5. Limitations

While this research broadly agrees that circulating nucleic acids hold great promise for improving IA diagnosis and prognostication, the field is still in its infancy, and several controversies and inconsistencies still need to be addressed. Different studies have sometimes reported conflicting results for the same biomarker. This discrepancy may stem from differences in the biological source analyzed, patient populations, or disease stage. Similarly, despite many studies identifying overlaps, the direction and magnitude of change varied across cohorts. To date, no RNA biomarker by itself has consistently proven to be a reliable stand-alone marker for IAs on which screening could be based.

Technical and biological challenges also temper the enthusiasm for clinical translation of these biomarkers. On the technical side, issues of sample handling and assay sensitivity are significant. RNAs, especially lncRNAs, can be prone to degradation. Additionally, differences in how blood is collected, processed (plasma vs. serum), and stored can lead to variability in results. Small RNA profiling methods (qRT-PCR arrays, next-generation sequencing) may have different biases and detection limits, complicating comparisons between studies. Future studies should incorporate harmonized preanalytical protocols and standardized detection platforms, as methodological differences remain one of the main drivers of inconsistency across the literature.

Moreover, most studies to date have small sample sizes, raising concerns about statistical power and potential selection bias. As noted in recent reviews, validation in larger independent cohorts is essential to confirm the diagnostic accuracy and robustness of proposed biomarkers. Thus, the major limitation of the available literature is the substantial heterogeneity in study design, including differences in biofluid source and patient populations, which complicates direct comparison across studies and weakens the strength of pooled conclusions.

Nonetheless, many circulating nucleic acids come from general processes like inflammation or tissue damage, so they are often not specific to IAs. For example, miR-21 and miR-146a are elevated not only in IA but also in other cardiovascular and neuroinflammatory conditions [68,69,70]. Therefore, an aneurysm may be indicated by a high level of a single circulating molecule, but other comorbid conditions may also be the cause. The non-specific background noise in these assays could lead to false positives if used for population screening. This limitation underscores that single RNA markers are unlikely to provide sufficient diagnostic accuracy on their own. Instead, multi-marker panels integrating several RNAs, ideally combined with imaging and clinical risk scores, may improve specificity and reduce false positives.

Inter-patient heterogeneity is another biological concern. IAs may occur in conjunction with various risk factors such as smoking, high blood pressure, and genetic predisposition, which have their own effects on gene expression profiles. For instance, because smokers are more likely to develop IA, smoking is known to change the circulating miRNA profiles, which could complicate biomarker analysis. A biomarker with an elevated IA could simply indicate endothelial stress caused by hypertension or smoking exposure. Carefully matched case-control studies and patient stratification by demographics and risk profiles in analyses are necessary to break down these factors.

Lastly, the fact that these biomarkers are still far from being routinely used in clinical settings is a practical limitation. Assays for circulating miRNAs for IAs are not yet standardized or accessible, in contrast to cholesterol or C-reactive protein testing already used for cardiovascular diseases and inflammatory processes. Developing cost-effective, rapid tests, perhaps using point-of-care microfluidic devices or CRISPR-based detection, will be necessary to translate this research into a screening tool that neurosurgeons or neurologists can readily deploy [71].

Despite these challenges, the substantial progress in the past few years gives reason for optimism. The field is moving toward consensus that liquid biopsy for IA is feasible, but continued efforts are needed to resolve current discrepancies and limitations.

## 5. Conclusions

Liquid biopsy biomarkers, including exosomal and circulating miRNAs, lncRNAs, and circRNAs, represent promising candidates for the detection, monitoring, and risk stratification of intracranial aneurysm formation, progression, and rupture. Future efforts should focus on the development of standardized biomarker panels, longitudinal studies assessing their predictive value, and the integration of molecular data with radiological and hemodynamic parameters to achieve more precise rupture risk stratification. Nonetheless, large-scale, multicenter validation studies remain indispensable before these biomarkers can be reliably translated into routine clinical practice.

## Figures and Tables

**Table 1:** Human studies evaluating liquid biopsy biomarkers in intracranial aneurysm patients.

Author, Year	Biomarker	Type	N. IA	IA vs. Control	Rupt vs. Non-Rupt	Source
Jin, 2013 [18]	miR-574	miRNA	24	↓		Plasma
	miR-22	miRNA	24	↑		Plasma
	miR-21	miRNA	24	↑		Serum
	miR-671-5p	miRNA	24	↑		Plasma
	miR-720	miRNA	24	↑		Plasma
	miR-936	miRNA	24	↓		Plasma
	miR-365	miRNA	24	↓		Plasma
	miR-498	miRNA	24	↑		Plasma
	miR-106b	miRNA	24	↑		Plasma
Li, 2014 [19]	miR-16	miRNA	40	↑		Plasma
	miR-25	miRNA	40	↑		Plasma
Su, 2015 [20]	miR-132	miRNA	58	↑		Plasma
	miR-324	miRNA	58	↑		Plasma
Zhong, 2019 [21]	miR-205	miRNA	91	↑		Plasma/Blood
Wang, 2016 [22]	miR-29a-3p	Exosomal miRNA	165	↓	↓	Plasma
Meeuwsen, 2017 [23]	miR-200a-3p	miRNA	15		↑	Serum
	miR-183-5p	miRNA	15	↓	↓	Serum
	miR-let7p-5p	miRNA	40	↓		Serum
Teng, 2017 [24]	circ_0021001	CircRNA	223	↓		Serum
Lai, 2017 [25]	miR-502-5p	miRNA	60	↑		Serum
	miR-1297	miRNA	60	↑		Serum
	miR-4320	miRNA	60	↑		Serum
Kikkawa, 2017 [26]	miR-6724	miRNA	10		↑	Plasma/CSF
	miR-15a	miRNA	10		↑	Plasma/CSF
Stylli, 2017 [27]	miR-451a	miRNA	20		↑	CSF
Sima, 2017 [28]	miR-145 (*)	miRNA	60	↓		Plasma
	miR-143-5p	miRNA	60	↓		Plasma
Ouyang, 2017 [29]	MALAT1	lncRNA	105	↑	↑	Blood
Lopes, 2018 [30]	miR-let-7f-5p	miRNA	30		↑	Serum
	miR-486-5p	miRNA	30		↓	Blood
	miR-126-5p	miRNA	30		↓	Blood
	miR-17-5p	miRNA	30		↓	Blood
	miR-451a	miRNA	30		↓	Blood
	miR-146a-5p	miRNA	30		↑	Blood
	miR-589-5p	miRNA	30		↑	Blood
	miR-941	miRNA	30		↑	Blood
Sheng, 2018 (1) [31]	miR-1297	miRNA	128		↑	Serum
Sheng, 2018 (2) [32]	miR-502-5p	miRNA	129		↑	Serum
Feng, 2018 [33]	miR-143	miRNA	17	↓	↓	Plasma
	miR-145 (*)	miRNA	17	↓	↓	Plasma
Xu, 2018 [34]	miR-145/miR-145-5p (*)	Exosomal miRNA	30	↓		Plasma
	miR-143 / 143-3p / 143-5p	miRNA	30	↓		Serum
Zhao, 2018 [35]	miR-29a-3p (*)	Exosomal miRNA	24	↑		Blood
Qin, 2019 [36]	miR-202-5p	Exosomal miRNA	NA		↑	Serum
Wu, 2019 [37]	TCONS_00000200	lncRNA	30	↑		Plasma
	ENST00000511927	lncRNA	30	↑		Plasma
Yang, 2019 [38]	miR-155	miRNA	94		↑	Blood
Man, 2019 [39]	GASL1	lncRNA	68	↓		Serum
Huang, 2019 [40]	circ_0072309	CircRNA	30	↓		Serum
	circ_0008433	CircRNA	30	↑		Serum
Zheng, 2020 [41]	miR-92a	miRNA	91	↑		Blood
	miR-21	miRNA	91	↑		Blood
Yang, 2020 [38]	miR-126	miRNA	102	↑		Serum
Supriya, 2020 [42]	miR-27b-3p	miRNA	88	↓		Serum
	miR-15a-5p	miRNA	88	↑		Serum
	miR-34a-5p (*)	miRNA	88	↑		Serum
	miR-374a-5p	miRNA	88	↑		Serum
	miR-146a-5p	miRNA	88	↓		Serum
	miR-376c-3p	miRNA	88	↓		Serum
	miR-18b-5p	miRNA	88	↓		Serum
	miR-24-3p	miRNA	88	↓		Serum
Liao, 2020 [43]	miR-145/miR-145-5p (*)	Exosomal miRNA	12	↑	↑	Plasma
	miR-29a-3p (*)	Exosomal miRNA	12	↑	↑	Plasma
Xu, 2021 [44]	HIF1A-AS1	lncRNA	56	↑		Blood
Wang, 2021 [45]	miR-29a-3p (*)	miRNA	165		↑	CSF
	Let-7b-5p	miRNA	31		↑	CSF
	miR-15b-5p	miRNA	31		↑	CSF
	miR-17-5p	miRNA	31		↑	CSF
	miR-19b-3p	miRNA	31		↑	CSF
	miR-20a-5p	miRNA	31		↑	CSF
	miR-24	miRNA	31		↑	CSF
Yang, 2021 [46]	miR-144-5p	Exosomal miRNA	12	↓		Serum
Yuan, 2021 [47]	miR-34a / 34a-5 (*)	miRNA	20	↓		Serum
Zheng, 2021 [48]	miR-513b-5p	miRNA	100	↓	↓	Serum
Jin, 2021 [49]	miRNA-21	miRNA	40	↑		Serum
Chen, 2021 [50]	circ_0005505	CircRNA	5		↓	Blood
Huang, 2021 [8]	circ_0008433	CircRNA	347		↓	Blood
	circ_0001946	CircRNA	347		↓	Blood
Wu, 2022 [51]	Circ_0007990	CircRNA	18	↑		Serum
Jiang, 2022 [52]	miRNA-1246	miRNA	58	↑		Blood
Xiong, 2022 [53]	miR-125a	miRNA	50	↑		Plasma
Li, 2022 [54]	miR-152-3p	miRNA	135	↓		CSF
Zheng, 2022 [55]	miR-23b-3p	miRNA	65		↓	Plasma
	miR-20b-5p	miRNA	65		↓	Plasma
	miR-590-5p	miRNA	65		↓	Plasma
	miR-142-3p	miRNA	65		↓	Plasma
	miR-29b-3p	miRNA	65		↓	Plasma
Huang, 2023 [56]	Circ_0000690	CircRNA	216	↓		Serum
Han, 2024 [57]	DUXZP8	lncRNA	312	↑		Serum
Zou, 2024 [58]	miR-34a / 34a-5 (*)	miRNA	20	↓		Serum
Deng, 2024 [59]	miR-140	miRNA	25	↑		Serum
Liu, 2025 [60]	MIAT	lncRNA	88	↑	↑	Blood
Chen, 2025 [61]	PTV1	lncRNA	90	↑		Serum
Ansari, 2025 [62]	miR-29a (*)	miRNA	24	↑	↑	Blood/serum/CSF
	miR-200a-3p	miRNA	24	↑	↑	Blood/serum/CSF
	miR-4	miRNA	24	↑	↑	Blood/serum/CSF

(*) Studies reporting discordant results; “↑”: plasmatic/serum level of miR increased; “↓”: plasmatic/serum level of miR decreased.

## Data Availability

Available upon reasonable request.

## Abbreviations

The following abbreviations are used in this manuscript:

ACMG	American College of Medical Genetics and Genomics
AUC	Area under the curve
cfDNA	Cell-free DNA
circRNA	Circular RNA
CSF	Cerebrospinal fluid
CT	Computed tomography
ECM	Extracellular matrix
EV	Extracellular vesicle
GCS	Glasgow Coma Scale
HHT	Hereditary hemorrhagic telangiectasia
IAs	Intracranial aneurysms
IRB	Institutional Review Board
ISAT	International Subarachnoid Aneurysm Trial
ISUIA	International Study of Unruptured Intracranial Aneurysms
LB	Liquid biopsy
lncRNA	Long non-coding RNA
MRI	Magnetic resonance imaging
PHASES	Population, Hypertension, Age, Size of aneurysm, Earlier subarachnoid hemorrhage, and Site of aneurysm
PRISMA	Preferred Reporting Items for Systematic Reviews and Meta-Analyses
qRT-PCR	Quantitative reverse transcription polymerase chain reaction
ROBINS-I	Risk Of Bias In Non-randomized Studies of Interventions
ROC	Receiver operating characteristic
RNA	Ribonucleic acid
SAH	Subarachnoid hemorrhage
UIA	Unruptured intracranial aneurysm
UIATS	Unruptured Intracranial Aneurysm Treatment Score
VSMC	Vascular smooth muscle cell
